# Appropriate use of tissue sampling and somatostatin receptor PET imaging in the diagnosis of pancreatic neuroendocrine tumors: results of an International Delphi Consensus

**DOI:** 10.1007/s00464-025-11667-8

**Published:** 2025-05-02

**Authors:** Megan Casey, Francesca Tozzi, Jaeyun Wang, Keon Min Park, Emily Bergsland, Thomas Hope, Hagen F. Kennecke, J. Bart Rose, Michele Babicky, Shayan S. Irani, Kevin M. El-Hayek, Mohammad Abu Hilal, Horacio J. Asbun, Sean Cleary, Peter Smeets, Frederik Berrevoet, Mohamed Adam, Niki Rashidian, Adnan Alseidi, PNET Diagnosis Research Group, Aman Chauhan, Andrew M. Bellizzi, Åsmund A. Fretland, Brendan C. Visser, Bryson W. Katona, Daniel Halperin, Delphine L. Chen, Eric Nakakura, Erik Mittra, Flavio G. Rocha, Grace E. Kim, Heloisa Soares, Anne Hoorens, Jess Maxwell, Jonathan M. Loree, Karen Geboes, Linda Lee, Michael H. Larsen, Monica Dua, Mustafa Arain, Nadine Mallak, Nancy Joseph, Nitya Raj, Pieter Hindryckx, Sun-Chuan Dai, Thorvardur R. Halfdanarson, Bliede Van den Broeck

**Affiliations:** 1 Department of Surgery, University of California San Francisco, San Francisco, USA; 2 Department of General, Hepatopancreaticobiliary Surgery and Liver Transplantation, Ghent University Hospital, Ghent, Belgium; 3 Department of Medicine, Division of Hematology Oncology, San Francisco (UCSF) and UCSF Helen Diller Family Comprehensive Cancer Center, University of California, San Francisco, USA; 4 Department of Radiology and Biomedical Imaging, University of California, San Francisco, USA; 5 Providence Cancer Institute, Portland, USA; 6 Division of Surgical Oncology, University of Alabama, Birmingham, USA; 7 Providence Portland Medical Center, The Oregon Clinic, Portland, USA; 8 Division of Gastroenterology and Hepatology, Virginia Mason Medical Centre, Seattle, USA; 9 Department of Surgery, The MetroHealth System, Case Western Reserve University School of Medicine, Cleveland, USA; 10 Department of Surgery, University of Jordan, Amman, Jordan; 11 Division of Hepatobiliary and Pancreas Surgery, Miami Cancer Institute, Miami, USA; 12 Division of General Surgery, University of Toronto, Toronto, Canada; 13 Department of Radiology, Ghent University Hospital, Ghent, Belgium; 14 Division of Surgical Oncology, University of California San Francisco, San Francisco, USA; 15 Faculty of Medicine, Southampton University Hospital, Southampton, United Kingdom

**Keywords:** Pancreatic neuroendocrine tumors, Somatostatin receptor positron emission tomography, Tissue sampling, Expert consensus, Diagnostic protocol, Ultrasound-guided biopsy

## Abstract

**Background:**

Current guidelines lack clarity regarding the appropriate use of preoperative ultrasound-guided (EUS) biopsy and receptor positron emission tomography (SSTR PET) imaging for pancreatic neuroendocrine tumors (PNETs). This study aims to reach expert consensus on the optimal sequencing of SSTR PET and EUS biopsy in the diagnostic workup and management of patients with suspected PNETs.

**Methods:**

A three-round modified Delphi process was used. A multidisciplinary panel of experts was recruited via snowball sampling. A set of 22 baseline statements pertaining to diagnostic workup, imaging, and biopsy was developed based on literature review and feedback obtained through a focus group. Survey rounds were conducted electronically and anonymously. A panel of international experts was asked to indicate whether they agreed, disagreed, or lacked the appropriate background to answer each statement. Of the 55 experts invited, 38 (69%) accepted to participate. Consensus was achieved with > 80% agreement.

**Results:**

Response rates were 97%, 100%, and 100% in rounds 1, 2, and 3, respectively. Following rounds 1 and 2, 29 final statements achieved consensus in the following three domains: diagnostic workup (15 statements), imaging (nine statements), and tissue sampling (five statements). Cronbach’s alpha value, a measure of internal consistency, was 0.91 and 0.85 for rounds 1 and 2, respectively. The final set of statements achieved a 95% approval rate in round 3.

**Conclusion:**

This international Delphi study provides expert consensus-based guidance on the appropriate use of EUS biopsy in the diagnostic workup of PNETs in the era of SSTR PET imaging.

**Supplementary Information:**

The online version contains supplementary material available at 10.1007/s00464-025-11667-8.

Pancreatic neuroendocrine tumors (PNETs) encompass a heterogeneous group of neoplasms, constituting 1.3% of all pancreatic tumors, with increasing incidence and prevalence [1–5]. This rise is attributed to enhanced early detection, advances in imaging techniques, and greater clinical awareness, reflecting improved diagnostic capabilities [6, 7]. Current guidelines for managing PNETs recommend primary surgical resection or clinical surveillance based on functionality, tumor stage, and grading [8–11].

The diagnostic workup for PNETs includes conventional imaging modalities such as computed tomography (CT), magnetic resonance imaging, and endoscopic ultrasound (EUS) for both diagnosis and staging [8, 10, 12–14]. EUS, which is routinely integrated into their clinical workup, offers the potential for tissue sampling to differentiate tumors. However, EUS-guided biopsy is an invasive procedure that can potentially be associated with postprocedural complications. Although the incidence of these complications is low, they can result in delayed treatment and increased healthcare costs [15, 16].

In addition to traditional diagnostic strategies, somatostatin receptor positron emission tomography (SSTR PET) plays a vital role in detecting, staging, and treating PNETs [17–19]. Advances in SSTR PET have shown comparable efficacy to EUS-guided biopsy in diagnosing PNETs and predicting tumor grading [20, 21]. SSTR PET is safe and reproducible, showing potential benefits for specific clinical cases without requiring prior tissue sampling for treatment or follow-up [22–24].

Although these advancements exist, a recent analysis investigating clinical workup practices for PNETs in the United States has shown a notable increase in EUS-guided biopsy rates for resectable PNETs post-2016, despite FDA approval and availability of SSTR PET [25]. Alongside the notable increase in diagnosis and use of EUS-guided biopsy, studies have highlighted the need to improve standards of care in the management of NET patients [26, 27].

Current guidelines for PNETs recommend histological diagnosis through preoperative biopsy or resection specimens, advocating for the use of one of these diagnostic steps. However, they do not specify which patients might benefit more from SSTR PET versus EUS-guided biopsy [10, 24, 28–30].

Given the complexity and heterogeneity of PNETs, along with existing gaps in guidelines defining the use and sequence of diagnostic tools, identifying the optimal diagnostic workup is crucial for improving PNET management practices. The Delphi method is a validated approach that provides guidance by aggregating expert opinions and best practices on clinical questions in areas where evidence-based medicine is limited, and complex issues need resolution [31, 32].

Therefore, the aim of this study was to establish an international multidisciplinary expert consensus on the optimal utilization of tissue sampling and SSTR PET in diagnosing PNETs, employing a modified Delphi method. By providing clear and practical case scenarios, this consensus aims to enhance clinical decision-making, reduce unnecessary procedures, and potentially improve patient outcomes in the management of PNETs.

## Materials and methods

This modified Delphi process consisted of a 3-round web-based survey following the Conducting and Reporting Delphi Studies guidelines [32]. This study was determined to be exempt by the IRB as it did not involve patients.

### Initial survey design

In the conventional Delphi method, the expert panel is responsible for defining the first-round statements. A modified Delphi method was used, and an initial set of baseline statements was developed based on a literature review of the best available evidence, existing societal guidelines, and feedback obtained through a focus group [10, 25, 29, 30, 33, 34]. The four authors comprising the steering committee (M.C., F.T., N.R., A.A.) conducted the literature review to identify pertinent issues. Key aspects concerning the diagnostic workup, imaging, and biopsy process of PNET were identified, and initial statements were formulated. Subsequently, a focus group was convened comprising seven experts from relevant specialties, including two surgical oncologists, two medical oncologists, two radiology/nuclear medicine specialists, and one interventional gastroenterologist. The focus group members were presented with the baseline statements and typical clinical scenarios relevant to the workup of PNETs. They were invited to provide feedback and suggest additional considerations for the subsequent Delphi process. Finally, the steering committee created the survey containing 22 baseline statements for distribution to the expert panel.

### Expert panel recruitment

Potential expert panel members were selected through purposive sampling based on their involvement in NET professional societies, contributions to existing guidelines, NET literature, and clinical experience in diagnosing and treating patients with PNETs. Recognizing the interdisciplinary nature of care for PNET patients, it was aimed to recruit a diverse panel of experts across multiple specialties, including surgical oncology, medical oncology, gastroenterology, radiology, nuclear medicine, and pathology. A standardized recruitment letter was sent via email to all potential panelists. Participation in the study was voluntary and anonymous, with all participants providing written informed consent. While there is no consensus on Delphi panel size, previous studies have recommended a minimum of 10 panelists for homogenous groups and 30 panelists for heterogeneous groups [31, 35]. Therefore, this study aimed to recruit a diverse panel of 30 to 40 experts.

### Delphi methodology

Consensus was predefined as participant agreement greater than 80% and Cronbach’s alpha value of greater than 0.8, consistent with definitions used in previous Delphi studies [35–38]. The surveys for each Delphi round were distributed via an online platform (Qualtrics XM). For each round, participants were given two weeks to respond. Participants who had not responded after two weeks received a reminder email, with a total response period of up to four weeks from initial survey distribution. Surveys were designed to include response requirements for each statement to prevent missing data.

**Fig. 1:**
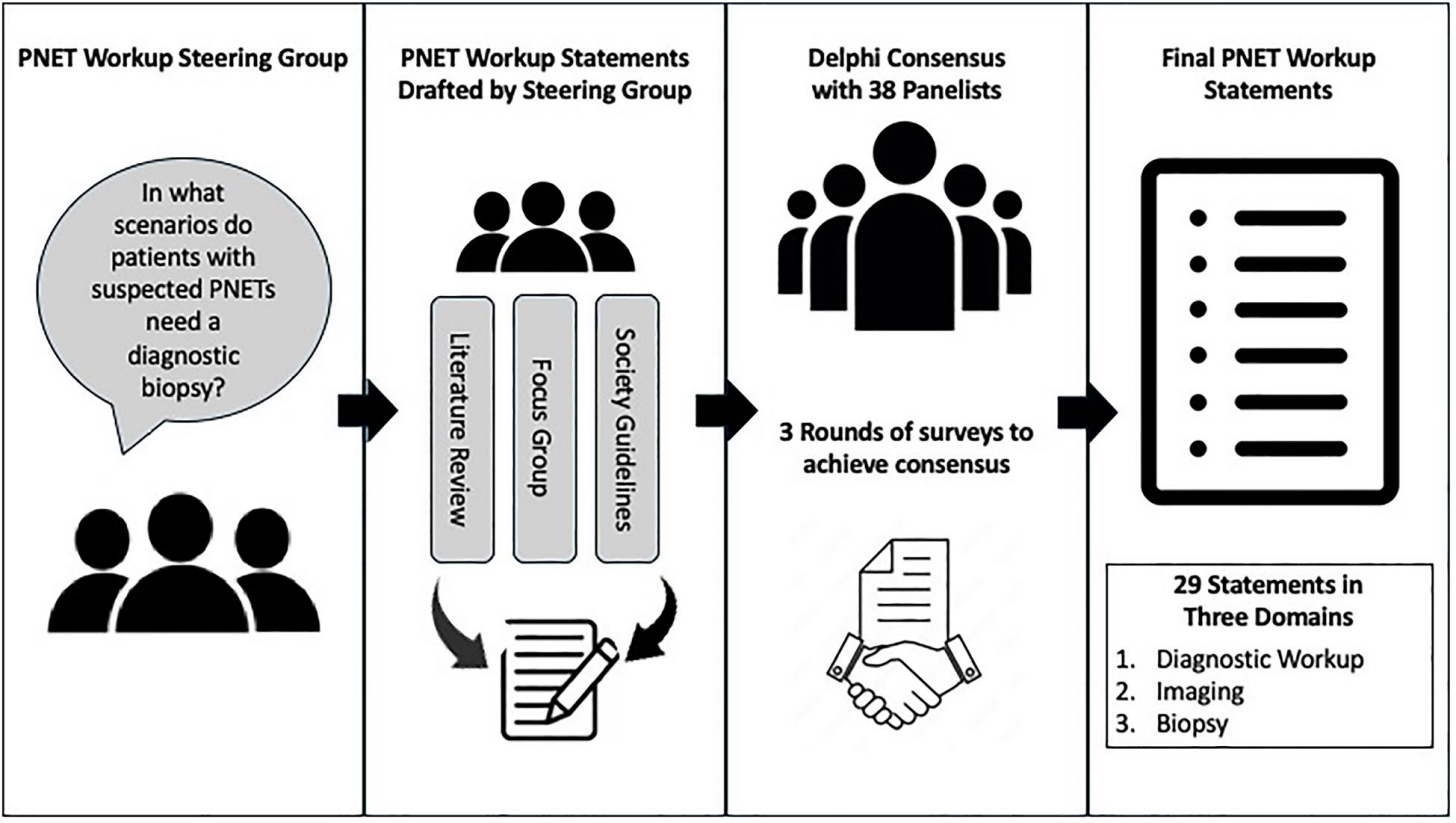
Summary of study design and Delphi process. *PNET* Pancreatic neuroendocrine tumor

The initial survey included 22 baseline statements in three different domains: diagnostic workup, imaging, and biopsy. Participants were asked to indicate whether they agreed or disagreed with a given statement. Given the multidisciplinary nature of the panel, participants were also given the option to indicate that a topic was beyond their practice’s scope and abstain from voting. Boxes for free-text responses were included in each survey subsection to encourage comments or suggestions. In instances where participants disagreed with a statement, mandatory feedback through the free-text box was required. After each voting round, the percentages of agreement were calculated and reported in the following round. Figure 1 demonstrates the summary of the study design and Delphi process.

Following the first round of voting, statements that achieved greater than 80% agreement were included in the final set of consensus statements and excluded the second-round survey. The remaining statements, which received less than 80% agreement were revised by the steering group based on panelists’ feedback. Additional statements were incorporated in response to comments received during the first round of voting. In the second round of voting, panelists were again asked to indicate their agreement or disagreement with each statement. Statements that achieved greater than 80% agreement were added to the final set of consensus statements, while those with less than 80% agreement were excluded.

The third round comprised the final set of proposed statements across the three domains. Panelists were asked to indicate their approval or disapproval of the full set of statements.

### Statistical analysis

Descriptive statistics were employed to summarize the results. Counts and percentages were used to report categorical variables, while continuous data for expert panel demographics were presented as median and range. The agreement rate among participants who did not opt out of voting was calculated for each statement in each round of the Delphi process. Cronbach’s alpha was used to determine internal consistency in the first two rounds, with a value above 0.7 indicating acceptable reliability and a value above 0.9 considered excellent. Statistical analyses were performed utilizing SPSS version 29.0 (IBM, Armonk, NY, USA).

## Results

The entire Delphi process occurred between June 2023 and April 2024. Out of a total of 55 invited experts, 38 (69%) agreed to participate in this study. The expert panel included surgical oncologists (*n* = 13), medical oncologists (*n* = 8), gastroenterologists (*n* = 8), pathologists (*n* = 4), nuclear medicine specialists (*n* = 4), and radiologists (*n* = 2). Most experts were from the United States and were affiliated with institutions that treated more than 20 patients diagnosed with PNETs annually. Characteristics of the panel members are detailed in Table 1.

**Table 1:** Characteristics of expert panel members

	N (%)
Total	38 (100)
Country	
United States	29 (76)
Belgium	5 (13)
Canada	2 (5)
Italy	1 (3)
Norway	1 (3)
Specialty	
Surgical Oncology	13 (34)
Medical Oncology	8 (21)
Gastroenterology	8 (21)
Pathology	4 (10)
Nuclear Medicine	3 (8)
Radiology	2 (5)
Practice Setting	
Academic	33 (87)
Private	3 (8)
Community	1 (3)
Federal/Government Hospital	1 (4)
Years in practice, mean (range)	13.4 (3–34)
Practice at High Volume Institution (> 20 PNET patients per year)?	
Yes	36 (95)
No	2 (5)

### First-round survey

The response rate was 97% for the first round, with a Cronbach’s alpha value of 0.91 indicating excellent internal consistency. Of the 22 baseline statements, 14 (63.6%) achieved greater than 80% agreement and were directly included in the final set of statements for round three. The remaining 8 baseline statements were revised for further consideration in the second round. In addition, nine new statements were added based on panelist suggestions to address gaps or clarify ambiguous areas. This resulted in 17 statements (eight revised and 9 new statements) for the second Delphi round. Original baseline statements, comments and first-round results are available in Supplementary Data S1.

### Second-round survey

The response rate was 100% with Cronbach’s alpha value of 0.85 for the second round. Of the 17 statements from the second round, 15 (88.2%) achieved greater than 80% agreement and were included in the final set of statements. The two remaining statements that did not achieve consensus had agreement rates of 65% and 70%, which were below the predefined threshold, and were removed from further rounds.

### Third-round survey

The response rate was 100% for the third round. Panelists cast a single vote to either approve or disapprove of the full set of statements, which consisted of the 29 final statements: 14 statements directly approved in Round 1 and 15 statements approved in Round 2. Approved diagnostic workup, imaging, and tissue sampling statements are shown in Tables 2, 3, and 4, respectively.

**Table 2:** Approved diagnostic workup statements

Diagnostic Workup 1: For a patient who is a surgical candidate with a > 2 cm pancreatic mass suspicious for nonfunctional PNET based on multiphase CT findings and initial laboratory evaluation:	Round	Consensus
Statement 1: Somatostatin Receptor (SSTR) PET imaging, if available, is the most appropriate next step for workup and staging	1	86%
Statement 2: If the mass is SSTR PET-avid without aggressive features or radiological features of high-grade nature, and there is no evidence of metastatic disease, the patient should proceed to surgical resection without preoperative tissue sampling	1	88%
Statement 3: Tissue sampling via EUS-FNA or FNB is warranted if there is concern for aggressive clinical or laboratory features, or radiological features of high-grade nature (i.e., low SUV max), or if there is a discrepancy between CT and SSTR PET findings	1	91%
Statement 4: If SSTR PET imaging demonstrates metastases, tissue sampling of the largest, most aggressive-appearing lesion with the lowest risk of tissue sampling-related complication should be performed to determine grade and possible genetic subtyping	1	86%

Diagnostic Workup 2: For a patient who is not a surgical candidate with a > 2 cm pancreatic mass suspicious for nonfunctional PNET based on multiphase CT findings and initial laboratory evaluation:	Round	Consensus
Statement 1: SSTR PET imaging should be obtained to determine extent of disease	1	89%
Statement 2: Tissue sampling should be performed to confirm the diagnosis and determine grade prior to initiating medical treatment	1	97%

Diagnostic Workup 3: For an asymptomatic patient with a ≤ 2 cm pancreatic mass suspicious for nonfunctional PNET based on multiphase CT findings and initial laboratory evaluation:	Round	Consensus
Statement 1: If there is no concern for metastases or high-grade features based on multiphasic CT/MRI findings, options include 1) short-term observation (especially for lesions < 1 cm); 2) SSTR PET imaging and 3) tissue sampling. This decision should be made based on discussions with the patient, lesion size, and available resources	2	89%
Statement 2: If the mass is SSTR-avid without aggressive features or radiological features of high-grade nature, and there is no evidence of metastatic disease, radiological observation with a follow-up multiphase CT or MRI is appropriate in lieu of immediate tissue sampling • *Comment 2a: For lesions greater than 1.5 cm that are SSTR-avid, surgical resection may be considered based on surgical risk/tumor location and discussion with the patient*	2 2	97% 92%
Statement 3: If the mass exhibits growth on surveillance imaging, the patient should be referred for surgical evaluation and discussed in a multidisciplinary tumor board. Tissue sampling to rule out high-grade differentiation (G3) and SSTR PET imaging to rule out occult metastatic disease not seen by conventional imaging should be considered	2	89%

Diagnostic Workup 4: For a patient who is a surgical candidate with a pancreatic mass suspicious for functional PNET based on multiphase CT findings and initial laboratory evaluation:	Round	Consensus
Statement 1: SSTR PET imaging is the most appropriate next step for workup and staging	1	88%
Statement 2: Tissue sampling is not required prior to surgical resection unless there is concern for aggressive features or radiological features of high-grade nature	1	88%

Diagnostic Workup 5: For a patient who is a surgical candidate with multifocal pancreatic masses and a clinical picture concerning for nonfunctional PNETs:	Round	Consensus
Statement 1: SSTR PET imaging, if available, is the appropriate next step for workup and staging	1	91%
Statement 2: If a specific lesion exhibits aggressive or high-grade features, EUS evaluation and tissue sampling of that lesion should be considered	1	88%
Statement 3: Germline genetic testing should be obtained to evaluate for MEN1 or other genetic syndromes depending on the clinical context	2	100%

**Table 3:** Approved imaging statements

Final imaging statements	Round	Consensus
Statement 1: Chest imaging is low yield for patients with small tumors and those with no evidence of hepatic or other abdominal metastases	2	94%
Statement 2: Low SUV max and poor correlation with multiphase CT findings are features on SSTR PET imaging that are concerning for high-grade PNET. This should prompt tissue sampling of the mass and possible FDG PET/CT for further evaluation of the lesion • *Comment 2a: If both FDG PET and tissue sampling are deemed necessary for a suspected PNET, FDG PET should be performed before tissue sampling to avoid false positives due to biopsy-related inflammation and to help guide the choice of lesion to biopsy in the case of multiple lesions*	1 2	97% 88%
Statement 3: For patients with pancreatic tail lesions that are suspicious for accessory spleen on multiphase CT and that may be SSTR-avid, a heat-damaged RBC scan, abdominal MRI with and without IV contrast, or EUS with or without tissue sampling should be considered to further characterize the lesion • *Comment 3a: In centers with significant experience, EUS without tissue sampling may help differentiate between a PNET and accessory spleen*	2 2	84% 81%
Statement 4: If SSTR PET imaging is u navailable, one should consider either referral to a center with SSTR PET imaging capability or an EUS to characterize suspected PNETs	2	86%
Statement 5: If SSTR PET imaging demonstrates uptake in the uncinate process of unclear etiology, or if the location of uptake is unclear (pancreas versus small bowel versus lymph node), EUS with or without tissue sampling or multiphase MRI can be used to further characterize the region of uncertainty • *Comment 5a: Tissue sampling *via* EUS should be done if indicated. However, EUS without tissue sampling also has a role in characterizing the lesion*	1 2	82% 81%
Statement 6: In situations where concern for multifocality exists, it is reasonable to obtain an EUS without tissue sampling prior to resection of suspected PNET to assess for additional lesions not seen on cross-sectional imaging	2	85%

**Table 4:** Approved tissue sampling statements

Final tissue sampling statements	Round	Consensus
Statement 1: If tissue sampling of a suspected PNET is deemed necessary, fine needle biopsy should be performed over fine needle aspiration to ensure adequate tissue sampling and avoid sampling error	1	87%
Statement 2: In-room cytology evaluation, if available, should be performed for EUS biopsies of suspected PNETs to ensure adequate sampling • *Comment 2a: If available, in-room cytology is strongly recommended for difficult-to-biopsy lesions or for cases that involve re-do tissue sampling*	2 2	81% 91%
Statement 3: When obtaining tissue, tissue sampling of an abnormal lymph node in lieu of the pancreatic mass is acceptable if deemed to be lower risk and in-room cytology is present to confirm the diagnosis	2	91%
Statement 4: A patient with a hypervascular pancreatic mass on multiphase CT and a history of another malignancy with the potential for hypervascular metastases (ex. renal cell carcinoma, hepatocellular carcinoma) should undergo tissue sampling of the pancreatic lesion to distinguish primary PNET from metastasis	1	94%

**Fig. 2:**
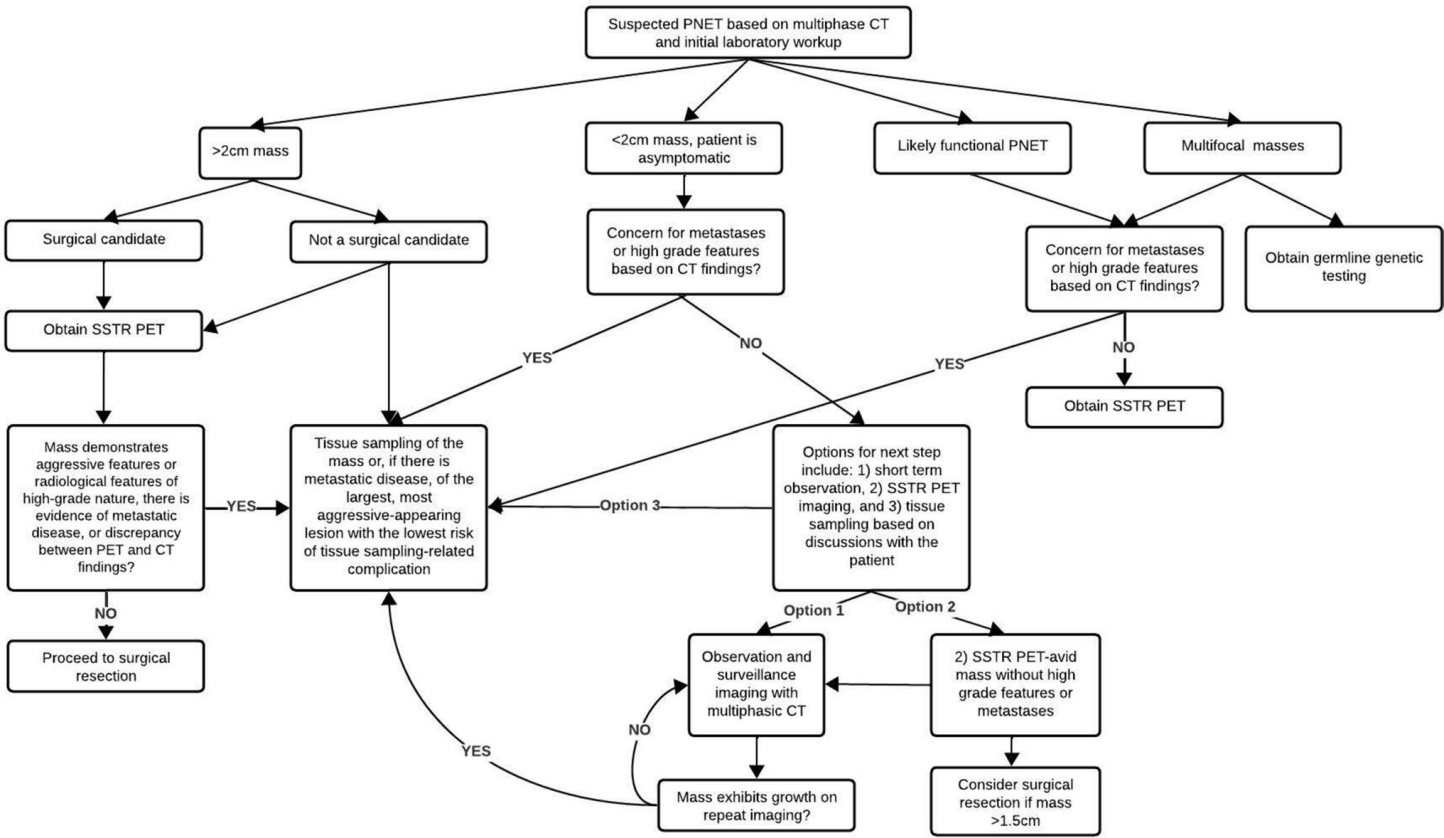
Flow chart for the diagnostic workup of pancreatic neuroendocrine tumors. *PNET* Pancreatic neuroendocrine tumor, *CT* Computed tomography, *SSTR PET* Somatostatin receptor positron emission tomography, *PET* Positron emission tomography

The final set of consensus statements achieved a 95% approval rate, with 2 (5.3%) out of 38 panelists voting to disapprove the set. Among the two panelists who voted to disapprove, one disagreed with two specific tissue sampling statements and suggested additional phrasing for one imaging statement. The other panelist disagreed with the phrasing of one imaging statement. Based on the clinical scenario-based statement, a detailed flow-chart for the diagnostic workup of PNET was developed (Fig. 2), while a summary of recommendations is presented in Table 5.

**Table 5:** Summary of recommendations for pancreatic neuroendocrine tumors

**Surgical candidates with > 2 cm nonfunctional PNET**
• Utilize SSTR PET imaging for workup and staging • Proceed to surgical resection without preoperative tissue sampling if the mass is SSTR PET-avid, lacks aggressive features, and shows no metastasis • Consider tissue sampling only if there are imaging discrepancies or suspicion of aggressive or high-grade radiological features • If metastases are detected on SSTR PET, sample the most aggressive-appearing lesions with the lowest risk of complications
**Non-surgical candidates with > 2 cm nonfunctional PNET**
• Utilize SSTR PET to evaluate disease extent • Perform tissue sampling for definitive diagnosis and grading before initiating medical treatment
**Asymptomatic patients with ≤ 2 cm nonfunctional PNET**
• Options include short-term observation, SSTR PET imaging, or selective tissue sampling based on lesion characteristics (size and clinical features), patient discussion, and available resources • If SSTR-avid without aggressive features or metastasis, use multiphase CT or MRI for follow-up instead of immediate tissue sampling • Consider surgical resection for larger (> 1.5 cm) SSTR-avid lesions, based on surgical risk/tumor location and discussion with the patient • Refer for surgical and multidisciplinary evaluation if growth is detected during follow-up. Perform tissue sampling to exclude high-grade (G3) tumors and use SSTR PET to assess occult metastatic disease
**Surgical candidates with functional PNET**
• Utilize SSTR PET imaging for workup and staging • Consider tissue sampling before surgical intervention in the presence of aggressive or high-grade radiological features
**Surgical candidates with multifocal PNETs**
• Utilize SSTR PET imaging for workup and staging • Consider tissue sampling before surgical resection in the presence of aggressive or high-grade radiological features • Obtain germline genetic testing to evaluate for MEN1 or other syndromes
**Imaging**
• Prefer SSTR PET and multiphase CT/MRI for primary imaging • Use prompt tissue sampling and possibly FDG PET/CT for suspected high-grade PNETs (e.g., low SUV max or poor correlation with multiphase CT) • Perform abdominal MRI or EUS (with or without tissue sampling) for tail lesions suspicious for accessory spleen or uncinate process lesions with unclear etiology or uptake location • Consider EUS without tissue sampling prior to resection when multifocality is suspected to assess for additional lesions not seen on cross-sectional imaging
**Tissue Sampling**
• Opt for fine needle biopsy over aspiration to minimize sampling error • Utilize in-room cytology, if available, particularly for difficult-to-biopsy lesions or re-dos to ensure adequate sampling • Consider lymph node sampling (if abnormal) instead of mass sampling if in-room cytology is present • Perform tissue sampling for hypervascular pancreatic masses or history of other malignancies to distinguish primary PNETs from metastases

## Discussion

This Delphi study leveraged the expertise of a multidisciplinary panel of physicians in diagnosing and treating patients with PNETs to define best practices for the use of tissue sampling and SSTR PET in the workup of these tumors. Panelists approved a comprehensive set of 29 statements regarding common clinical workup scenarios, SSTR PET, and tissue sampling.

Although clinical practice should be individualized based on the unique characteristics of each patient, this Delphi consensus study could provide a valuable framework to guide decision making in the workup of suspected PNETs. The rising incidence of PNETs [1] and increased preoperative tissue sampling, despite advancements in imaging modalities[25], may benefit from better defined workup recommendations.

Existing guidelines pertaining to the workup of patients with suspected PNETs comment on the use of SSTR PET imaging as well as biopsy but frequently fail to provide specific suggestions regarding prioritization of these two diagnostic testing modalities in different clinical contexts. For example, published European guidelines state that SSTR PET should be part of the staging and preoperative workup for patients with suspected PNETs. These guidelines also indicate that histological diagnosis, while mandatory for all patients with PNETs, can be established using either resection specimens or core biopsy specimens [10]. This suggests that a definitive histological diagnosis is not necessary prior to surgical resection, aligning with the results of this Delphi. However, these guidelines do not explicitly specify the clinical scenarios in which this approach is appropriate, nor the recommended sequence of the diagnostic workup involving tissue sampling and SSTR PET. Other published guidelines similarly use non-specific language [29, 30, 33, 34]. The consensus statements from the current study agree with existing guidelines, yet they provide additional guidance on prioritizing diagnostic modalities and integrating their findings in specific clinical contexts.

In the current study, consensus was achieved for specific clinical scenarios: surgical candidates with large (> 2 cm) pancreatic masses concerning for PNET, masses suspicious for functional PNETs, or multifocal pancreatic masses concerning for PNETs. For these cases, SSTR PET is favored over tissue sampling as the initial targeted diagnostic step. Furthermore, panelists agreed that tissue sampling of suspected PNETs following SSTR PET imaging was deemed unnecessary prior to surgical resection in surgical candidates with > 2 cm SSTR PET-avid tumors without evidence of metastatic disease, aggressive features or radiological features of high-grade nature. The 2 cm cutoff chosen in the diagnostic workup scenarios is consistent with previous literature in the field, including findings from the ASPEN and PANDORA prospective observation studies, although there is ongoing discussion regarding the appropriate management of < 2 cm PNETs [39–43]. A recent multicenter study suggests that relying solely on the 2 cm cutoff may be inadequate. This study found that PNETs exhibit significant heterogeneity in malignancy, and therefore, treatment strategies should integrate tumor grading alongside size. [44] Further consensus was reached on scenarios warranting tissue sampling in addition to SSTR PET imaging. These include concerns for high-grade PNET, evidence of metastases, ineligibility for surgical resection, or inconclusive SSTR PET imaging. Tissue sampling prior to SSTR PET imaging was deemed appropriate in cases when a patient has a hypervascular pancreatic mass on CT and history of other hypervascular malignancy.

Previous studies have demonstrated the diagnostic superiority of EUS-guided biopsy over EUS-guided cytologic aspiration, suggesting that tissue sampling should become the standard of care, especially for small PNETs [45, 46]. Supporting this evidence, albeit limited, experts in this Delphi study reached a consensus that fine needle biopsy is preferable to fine needle aspiration. Furthermore, in-room cytology evaluation is strongly advocated to ensure adequate tissue sampling and mitigate sampling errors, specifically for difficult-to-biopsy lesions or for cases that involve re-do tissue sampling. Notably, an increasing body of literature shows improved diagnostic performance when cytology evaluation is performed on-site [47–49].

Of the 31 statements voted upon, only two statements did not achieve consensus. These statements pertained to chest imaging in the absence of SSTR PET imaging and the appropriate use of SSTR PET imaging for insulinomas. For PNETs, especially when small, chest imaging lacks sufficient accuracy to detect distant metastases, particularly small nodules. This aligns and further specifies with current guidelines, which do not standardly recommend chest imaging. Consequently, consideration on a case-by-case basis is advisable [29].

In the third round of voting to approve the complete set of 29 statements, two panelists voted to disapprove, citing issues primarily related to the phrasing of the statements. Although each participant’s input holds equal value, a Delphi study’s intrinsic strength lies in harnessing collective intelligence to address complex issues through consensus [31]. With this additional context, we remain confident that the complete set of 29 statements represents best practices in the diagnostic workup of suspected PNETs, endorsed by the knowledgeable multidisciplinary panel of experts.

There are several limitations to the current study that should be addressed. First, the Delphi methodology is subjective, and selection of expert panelists are prone to selection and volunteer biases. Second, we sought to recruit a diverse panel of experts from multiple specialties involved in the diagnostic workup and treatment of patients with PNETs; however, some specialties may have been underrepresented. Third, several opportunities were provided for panelists to introduce new topics and statements they found relevant, both in the pre-Delphi focus group as well as throughout voting rounds; it is possible, although unlikely, that a relevant topic or clinical scenario, could have been overlooked. Finally, the consensus statements were generated and approved by acting under the assumption that insurance status and approval or availability of the diagnostic tests should not factor into clinical decision-making. It is well known that insurance coverage, availability and cost can play a significant role in dictating diagnostic workup, especially in countries such as the United States, and therefore several of these identified best practices may be difficult to implement into the current healthcare system.

The results of this study have several implications for the diagnostic workup of patients with suspected PNETs. Precisely defining the appropriate use of SSTR PET imaging and tissue sampling in various clinical scenarios, as outlined by these consensus statements, could lead to more streamlined workup for patients, particularly those with localized resectable tumors who may be able to forego tissue sampling prior to surgical resection. This could help reduce the increasing rate of preoperative tissue sampling that has previously been reported along with the associated morbidity and procedural costs [25]. Clinicians with less experience in diagnosing and treating patients with suspected PNETs can refer to the statements generated in this study to better inform workup decisions for their patients.

In addition, this study employs a multidisciplinary approach to facilitate an integrated workup of PNETs, involving clinicians from diverse backgrounds. This could lead to more appropriate referrals to the relevant specialists and improve the time to treatment. Future directions include working to incorporate results from this Delphi consensus study with existing societal guidelines to enhance the uptake of recommendations in clinical practice.

In conclusion, using a modified Delphi method, an international multidisciplinary expert consensus has been established on the appropriate use of tissue sampling and SSTR PET imaging in the diagnostic workup of PNETs. The final set of statements of this study can align for potential integration with existing guidelines to further improve the diagnostic workup process for patients with PNETs.

## Supplementary Information

Below is the link to the electronic supplementary material.

Supplementary file1 (DOCX 27 KB)
