# Supplementary material for: Appropriate use of tissue sampling and somatostatin receptor PET imaging in the diagnosis of pancreatic neuroendocrine tumors: results of an International Delphi Consensus
Source: Surg Endosc. 2025 May 2;39(6):3435–46. doi: 10.1007/s00464-025-11667-8 (PMC12116717; doi:10.1007/s00464-025-11667-8)
Supplement: Supplementary file 1 — Supplementary file1 (DOCX 27 KB) [file 464_2025_11667_MOESM1_ESM.docx]

**Supplementary Data S1.** Original baseline statements and comments.

| **Subject** | **Statement/**  **Comment Number** | **Round 1 Version** | **Round 1** | | **Round 2 Version** | **Round 2** | | **Included in Final Consensus Statement?** | **Final Statement** |
| --- | --- | --- | --- | --- | --- | --- | --- | --- | --- |
|  |  |  | **Number voted** | **% Agreement** |  | **Number voted** | **% Agreement** |  |  |
|  |  |  |  |  |  |  |  |  |  |
| Diagnostic Workup 1: For a patient who is a surgical candidate with a >2cm pancreatic mass suspicious for nonfunctional PNET based on multiphase CT findings and initial laboratory evaluation: | Statement 1 | DOTATATE PET/CT, if available, is the most appropriate next step for workup and staging. | 35 | 85.71% | Somatostatin Receptor (SSTR) ~~DOTATATE~~ PET/CT or PET/MR, if available, is the most appropriate next step for workup and staging. | __ | __ | YES | Somatostatin Receptor (SSTR) PET imaging, if available, is the most appropriate next step for workup and staging. |
|  | Statement 2 | If the mass is DOTATATE-avid without aggressive features or radiological features of high-grade nature, and there is no evidence of metastatic disease, the patient should proceed to surgical resection without preoperative biopsy. | 35 | 87.88% | If the mass is SSTR PET~~DOTATATE~~-avid without aggressive features or radiological features of high-grade nature, and there is no evidence of metastatic disease, the patient should proceed to surgical resection without preoperative biopsy | __ | __ | YES | If the mass is SSTR PET-avid without aggressive features or radiological features of high-grade nature, and there is no evidence of metastatic disease, the patient should proceed to surgical resection without preoperative biopsy |
|  | Statement 3 | Biopsy is warranted if there is concern for aggressive features or radiological features of high-grade nature, or if there is a discrepancy between CT and DOTATATE PET findings. | 35 | 91.43% | ~~Biopsy~~ Tissue sampling via EUS-FNA or FNB is warranted if there is concern for aggressive features or radiological features of high-grade nature (i.e. low SUV max), or if there is a discrepancy between CT and DOTATATE PET findings. | __ | __ | YES | Tissue sampling via EUS-FNA or FNB is warranted if there is concern for aggressive features or radiological features of high-grade nature (i.e. low SUV max), or if there is a discrepancy between CT and SSTR PET findings. |
|  | Statement 4 | If DOTATATE PET/CT demonstrates metastases, biopsy of the least risky lesion should be performed to determine grade and genetic subtyping. | 35 | 85.71% | If SSTR ~~DOTATATE~~ PET/CT or PET/MR demonstrates metastases, tissue sampling ~~biopsy~~ of the largest, most aggressive-appearing lesion with the lowest risk of biopsy-related complication ~~least risky lesion~~ should be performed to determine grade and possible genetic subtyping. | __ | __ | YES | If SSTR PET imaging demonstrates metastases, tissue sampling of the largest, most aggressive-appearing lesion with the lowest risk of biopsy-related complication should be performed to determine grade and possible genetic subtyping. |
| Diagnostic Workup 2: For a patient who is *not* a surgical candidate with a >2cm pancreatic mass suspicious for nonfunctional PNET based on multiphase CT findings and initial laboratory evaluation: | Statement 1 | DOTATATE PET/CT, if available, should be obtained to determine extent of disease. | 35 | 88.57% | SSTR ~~DOTATATE~~ PET/CT or PET/MR~~, if available~~, should be obtained to determine extent of disease. | __ | __ | YES | SSTR PET imaging should be obtained to determine extent of disease. |
|  | Statement 2 | Biopsy should be performed to confirm diagnosis and determine grade and genetic subtyping prior to initiating medical treatment. | 35 | 97.14% | ~~Biopsy~~ Tissue sampling should be performed to confirm the diagnosis and determine grade ~~and genetic subtyping~~ prior to initiating medical treatment. | __ | __ | YES | Tissue sampling should be performed to confirm the diagnosis and determine grade prior to initiating medical treatment. |
| Diagnostic Workup 3: For an asymptomatic patient with a ≤2cm pancreatic mass suspicious for nonfunctional PNET based on multiphase CT findings and initial laboratory evaluation: | Statement 1 | NANETS guidelines recommend that, if the patient is asymptomatic and there is no concern for metastases or high-grade features based on CT/MRI findings, radiological observation with a follow-up CT or MRI in 6 months is appropriate in lieu of immediate DOTATATE PET/CT or biopsy. | 32 | 71.88% | ~~NANETS guidelines recommend that,~~ If no concern for metastases or high-grade features based on multiphasic CT/MRI findings, ~~radiological observation with a follow-up CT or MRI in 6 months is appropriate in lieu of immediate DOTATATE PET/CT or biopsy~~ options include: short term observation (especially for lesions <1cm) vs initial SSTR PET/CT or PET/MR vs biopsy based on discussions with patient, size of lesion, and resources available. | 36 | 88.89% | YES | If there is no concern for metastases or high-grade features based on multiphasic CT/MRI findings options include: 1) short term observation (especially for lesions <1cm) 2) initial SSTR PET imaging or 3) tissue sampling based on discussions with the patient, size of lesion, and resources available. |
|  | Statement 2 | If the mass exhibits rapid growth on surveillance imaging, DOTATATE PET/CT, if available, should be obtained. | 33 | 78.79% | ~~If the mass exhibits rapid growth on surveillance imaging, DOTATATE PET/CT, if available, should be obtained.~~ If the mass is SSTR-avid without aggressive features or radiological features of high-grade nature, and there is no evidence of metastatic disease, radiological observation with a follow-up CT or MRI in 6 months is appropriate in lieu of immediate tissue sampling. | 36 | 97.22% | YES | If the mass is SSTR-avid without aggressive features or radiological features of high-grade nature, and there is no evidence of metastatic disease, radiological observation with a follow-up multiphase CT or MRI in 6 months is appropriate in lieu of immediate tissue sampling. |
|  | Comment 2a |  | __ | __ | For lesions greater than 1.5cm that are SSTR-avid, there may be a discussion of surgical resection based on surgical risk/tumor location and discussion with the patient. | 36 | 91.67% | YES | For lesions greater than 1.5cm that are SSTR-avid, surgical resection may be considered based on surgical risk/tumor location and discussion with the patient. |
|  | Statement 3 | If the mass exhibits rapid growth on surveillance imaging, a biopsy should be obtained. | 34 | 76.47% | If the mass exhibits ~~rapid~~ growth on surveillance imaging patients should be referred for surgical evaluation and discussed in a multidisciplinary tumor board. Tissue sampling to rule out high grade differentiation (G3/G4) and SSTR imaging to rule out occult metastatic disease not seen by conventional imaging should be considered. | 36 | 88.89% | YES | If the mass exhibits growth on surveillance imaging the patient should be referred for surgical evaluation and discussed in a multidisciplinary tumor board. Tissue sampling to rule out high grade differentiation (G3/G4) and SSTR PET imaging to rule out occult metastatic disease not seen by conventional imaging should be considered. |
| Diagnostic Workup 4: For a patient who is a surgical candidate with a pancreatic mass suspicious for functional PNET based on multiphase CT findings and initial laboratory evaluation: | Statement 1 | DOTATATE PET/CT, if available, is the most appropriate next step for workup and staging. | 34 | 88.24% | ~~DOTATATE~~ SSTR PET/CT or PET/MR~~, if available~~, is the most appropriate next step for workup and staging. | __ | __ | YES | SSTR PET imaging is the most appropriate next step for workup and staging. |
|  | Comment 1a |  | __ | __ | SSTR PET/CT or PET/MR may not be indicated in insulinomas since the majority are benign and lack sufficient SSTR expression. | 31 | 70.97% | NO | __ |
|  | Statement 2 | EUS biopsy is not required prior to surgical resection unless there is concern for aggressive features or radiological features of high-grade nature. | 32 | 87.50% | SSTR PET/CT or PET/MR may not be indicated in insulinomas since the majority are benign and lack sufficient SSTR expression. | __ | __ | YES | Tissue sampling ~~EUS biopsy~~ is not required prior to surgical resection unless there is concern for aggressive features or radiological features of high-grade nature. |
| Diagnostic Workup 5: For a patient who is a surgical candidate with multifocal pancreatic masses and a clinical picture concerning for nonfunctional PNETs**:** | Statement 1 | DOTATATE PET/CT, if available, is the appropriate next step for workup and staging. | 34 | 91.18% | ~~DOTATATE~~ SSTR PET/CT or PET/MR~~, if available~~, is the appropriate next step for workup and staging. | __ | __ | YES | SSTR PET imaging is the appropriate next step for workup and staging. |
|  | Statement 2 | If a specific lesion exhibits aggressive or high-grade features, biopsy of that lesion should be considered. | 34 | 88.24% | If a specific lesion exhibits aggressive or high-grade features, ~~biopsy~~ EUS evaluation and tissue sampling of that lesion should be considered. | __ | __ | YES | If a specific lesion exhibits aggressive or high-grade features, EUS evaluation and tissue sampling of that lesion should be considered. |
|  | Statement 3 |  | __ | __ | Genetic testing should be obtained to evaluate for MEN or other genetic syndromes depending on the clinical context. | 38 | 100.00% | YES | Germline genetic testing should be obtained to evaluate for MEN or other genetic syndromes depending on the clinical context. |
| Imaging | Statement 1 | Chest imaging should be obtained for patients with suspected PNET if they do not undergo DOTATATE PET/CT. | 35 | 74.29% | Chest imaging should be obtained for patients with suspected PNET if they do not undergo SSTR ~~DOTATATE~~ PET/CT or PET/MR. | 32 | 65.63% | NO | __ |
|  | Comment 1a |  | __ | __ | Chest imaging is likely low-yield for small tumors and in patients with no evidence of hepatic or other abdominal metastases. | 32 | 93.75% | YES | Chest imaging is low-yield for patients with small tumors and in those with no evidence of hepatic or other abdominal metastases. |
|  | Statement 2 | Low SUV max and poor correlation with high resolution CT findings are features on DOTATATE PET/CT that are concerning for high-grade PNET and should prompt biopsy of the mass and possibly FDG PET/CT for evaluation of metastatic disease. | 29 | 96.55% | Low SUV max and poor correlation with high resolution CT findings are features on SSTR ~~DOTATATE~~ PET/CT or PET/MR that are concerning for high-grade PNET and should prompt tissue sampling ~~biopsy~~ of the mass and possibly FDG PET/CT for evaluation of metastatic disease. | __ | __ | YES | Low SUV max and poor multiphase CT findings are features on SSTR PET imaging that are concerning for high-grade PNET and should prompt tissue sampling of the mass and possibly FDG PET/CT for evaluation of the lesion. |
|  | Comment 2a |  | __ | __ | If both FDG PET and tissue sampling are deemed necessary for a suspected PNET, FDG PET should be performed before tissue sampling to avoid false positives due to biopsy-related inflammation. | 34 | 88.24% | YES | If both FDG PET and tissue sampling are deemed necessary for a suspected PNET, FDG PET should be performed before tissue sampling to avoid false positives due to biopsy-related inflammation and to help guide the choice of lesion to biopsy. |
|  | Statement 3 | EUS without biopsy can be used to characterize DOTATATE-avid pancreatic tail masses to distinguish PNET from possible accessory spleen prior to treatment decision-making. | 29 | 58.62% | ~~EUS without biopsy~~ Patients with pancreatic tail lesions that are, on multiphase CT, suspicious for accessory spleen and that may be SSTR-avid, should undergo an RBC scan or diffusion-weighted MRI to further characterize the lesion. | 32 | 84.38% | YES | For patients with pancreatic tail lesions that are, on multiphase CT, suspicious for accessory spleen and that may be SSTR-avid, a heat-damaged RBC scan, diffusion-weighted MRI or EUS with or without tissue sampling shouldb be considered to further characterize the lesion. |
|  | Comment 3a |  | __ | __ | In centers with significant experience, EUS without biopsy may help differentiate between PNET and a splenule. | 32 | 81.25% | YES | In centers with significant experience, EUS without biopsy may help differentiate between PNET and an accessory spleen. |
|  | Statement 4 | If DOTATATE PET/CT is unavailable, high-resolution MRI or EUS without biopsy can be used to characterize suspected PNETs. | 32 | 59.38% | If SSTR ~~DOTATATE~~ PET/CT or PET/MR is unavailable, ~~high-resolution MRI~~ one should consider either referral to a center with SSTR PET imaging capability or an EUS ~~without biopsy~~ to characterize suspected PNETs. | 36 | 86.11% | YES | If SSTR PET imaging is unavailable, one should consider either referral to a center with SSTR PET imaging capability or an EUS to characterize suspected PNETs. |
|  | Statement 5 | If DOTATATE PET/CT demonstrates uncinate process uptake of unclear etiology, or if unclear whether DOTATATE PET/CT uptake is located in the pancreas versus small bowel versus lymph node, EUS without biopsy or MRI can be used to further characterize the lesion. | 33 | 81.82% | If SSTR ~~DOTATATE~~ PET/CT or PET/MR demonstrates uncinate process uptake of unclear etiology, or if unclear whether ~~DOTATATE~~ SSTR PET/CT or PET/MR uptake is located in the pancreas versus small bowel versus lymph node, EUS without biopsy or MRI can be used to further characterize the region of uncertainty ~~lesion~~. | __ | __ | YES | If SSTR PET imaging demonstrates uncinate process uptake of unclear etiology, or if the location of uptake is unclear (pancreas versus small bowel versus lymph node), EUS without biopsy or multiphase MRI can be used to further characterize the region of uncertainty. |
|  | Comment 5a |  | __ | __ | Tissue sampling via EUS should be done if indicated. It remains that EUS without biopsy, by itself, has a role to characterize the lesion. | 32 | 81.25% | YES | Tissue sampling via EUS should be done if indicated. It remains that EUS without biopsy, by itself, has a role to characterize the lesion. |
|  | Statement 6 |  | __ | __ | In situations where concern for multifocality exists, it is reasonable to obtain an EUS without tissue sampling prior to resection of suspected PNET to assess for additional lesions not seen on cross-sectional imaging. | 34 | 85.29% | YES | In situations where concern for multifocality exists, it is reasonable to obtain an EUS without tissue sampling prior to resection of suspected PNET to assess for additional lesions not seen on cross-sectional imaging. |
| Biopsy | Statement 1 | If EUS biopsy of a suspected PNET is deemed necessary, fine needle biopsy should be performed over fine needle aspiration to ensure adequate tissue sampling and avoid sampling error. **(86.7%)** | 30 | 86.67% | If ~~EUS biopsy~~ tissue sampling of a suspected PNET is deemed necessary, fine needle biopsy should be performed over fine needle aspiration to ensure adequate tissue sampling and avoid sampling error. | __ | __ | YES | If tissue sampling of a suspected PNET is deemed necessary, fine needle biopsy should be performed over fine needle aspiration to ensure adequate tissue sampling and avoid sampling error. |
|  | Statement 2 | In-room cytology evaluation should be performed for EUS biopsies of suspected PNETs to ensure adequate sampling. | 28 | 67.86% | In-room cytology evaluation, if available, should be performed for EUS biopsies of suspected PNETs to ensure adequate sampling | 32 | 81.25% | YES | In-room cytology evaluation, if available, should be performed for EUS biopsies of suspected PNETs to ensure adequate sampling |
|  | Comment 2a |  | __ | __ | In room cytology is strongly recommended in difficult to biopsy lesions or re-do tissue sampling | 32 | 90.63% | YES | If available, in room cytology is strongly recommended for difficult to biopsy lesions or for cases that involve re-do tissue sampling. |
|  | Statement 3 | If an abnormal lymph node is identified in addition to suspected PNET on imaging, biopsy of the lymph node should be performed since it is lower risk and provides both diagnostic and staging information. | 30 | 76.67% | When obtaining tissue, tissue sampling of an abnormal lymph node in lieu of the pancreatic mass is acceptable if deemed to be lower risk and in-room cytology is present to confirm the diagnosis. | 34 | 91.18% | YES | When obtaining tissue, tissue sampling of an abnormal lymph node in lieu of the pancreatic mass is acceptable if deemed to be lower risk and in-room cytology is present to confirm the diagnosis. |
|  | Statement 4 | A patient with a hypervascular pancreatic mass on CT and a history of another malignancy with the potential for hypervascular metastases (ex. renal cell carcinoma, hepatocellular carcinoma) should undergo EUS biopsy of the pancreatic lesion to distinguish primary PNET from metastasis. | 36 | 94.44% | A patient with a hypervascular pancreatic mass on CT and a history of another malignancy with the potential for hypervascular metastases (ex. renal cell carcinoma, hepatocellular carcinoma) should undergo ~~EUS biopsy~~ tissue sampling of the pancreatic lesion to distinguish primary PNET from metastasis. | __ | __ | YES | A patient with a hypervascular pancreatic mass on multiphase CT and a history of another malignancy with the potential for hypervascular metastases (ex. renal cell carcinoma, hepatocellular carcinoma) should undergo tissue sampling of the pancreatic lesion to distinguish primary PNET from metastasis. |
